# The Adenovirus E4-ORF3 Protein Stimulates SUMOylation of General Transcription Factor TFII-I to Direct Proteasomal Degradation

**DOI:** 10.1128/mBio.02184-15

**Published:** 2016-01-26

**Authors:** Rebecca G. Bridges, Sook-Young Sohn, Jordan Wright, Keith N. Leppard, Patrick Hearing

**Affiliations:** a Department of Molecular Genetics and Microbiology, School of Medicine, Stony Brook University, Stony Brook, New York, USA; b School of Life Sciences, University of Warwick, Coventry, United Kingdom

## Abstract

Modulation of host cell transcription, translation, and posttranslational modification processes is critical for the ability of many viruses to replicate efficiently within host cells. The human adenovirus (Ad) early region 4 open reading frame 3 (E4-ORF3) protein forms unique inclusions throughout the nuclei of infected cells and inhibits the antiviral Mre11-Rad50-Nbs1 DNA repair complex through relocalization. E4-ORF3 also induces SUMOylation of Mre11 and Nbs1. We recently identified additional cellular targets of E4-ORF3 and found that E4-ORF3 stimulates ubiquitin-like modification of 41 cellular proteins involved in a wide variety of processes. Among the proteins most abundantly modified in an E4-ORF3-dependent manner was the general transcription factor II–I (TFII-I). Analysis of Ad-infected cells revealed that E4-ORF3 induces TFII-I relocalization and SUMOylation early during infection. In the present study, we explored the relationship between E4-ORF3 and TFII-I. We found that Ad infection or ectopic E4-ORF3 expression leads to SUMOylation of TFII-I that precedes a rapid decline in TFII-I protein levels. We also show that E4-ORF3 is required for ubiquitination of TFII-I and subsequent proteasomal degradation. This is the first evidence that E4-ORF3 regulates ubiquitination. Interestingly, we found that E4-ORF3 modulation of TFII-I occurs in diverse cell types but only E4-ORF3 of Ad species C regulates TFII-I, providing critical insight into the mechanism by which E4-ORF3 targets TFII-I. Finally, we show that E4-ORF3 stimulates the activity of a TFII-I-repressed viral promoter during infection. Our results characterize a novel mechanism of TFII-I regulation by Ad and highlight how a viral protein can modulate a critical cellular transcription factor during infection.

## IMPORTANCE

Adenovirus has evolved a number of mechanisms to target host signaling pathways in order to optimize the cellular environment during infection. E4-ORF3 is a small viral protein made early during infection, and it is critical for inactivating host antiviral responses. In addition to its ability to capture and reorganize cellular proteins, E4-ORF3 also regulates posttranslational modifications of target proteins, but little is known about the functional consequences of these modifications. We recently identified TFII-I as a novel target of E4-ORF3 that is relocalized into dynamic E4-ORF3 nuclear structures and subjected to E4-ORF3-mediated SUMO modification. Here, we show that TFII-I is targeted by E4-ORF3 for ubiquitination and proteasomal degradation and that E4-ORF3 stimulates gene expression from a TFII-I-repressed viral promoter. Our findings suggest that the specific targeting of TFII-I by E4-ORF3 is a mechanism to inactivate its antiviral properties. These studies provide further insight into how E4-ORF3 functions to counteract host antiviral responses.

## INTRODUCTION

Adenoviruses (Ads) are ubiquitous pathogens infecting a wide range of vertebrates. Ad infection is generally associated with mild respiratory, ocular, and gastrointestinal diseases, but Ads have been recognized in recent years as significant pathogens in immunocompromised patients (1). Ad has evolved mechanisms to regulate and exploit diverse cellular pathways to ensure efficient replication of the viral genome and production of new virus. These include Ad modulation of the cell cycle, host gene expression, intrinsic cellular antiviral responses, and innate and acquired immune responses (2, 3). Ad early region 4 (E4) gene products contribute to the regulation of many of these processes (3, 4). The adenovirus type 5 (Ad5) E4 region encodes six separate proteins that promote cell proliferation, regulate apoptosis, and counteract intrinsic cellular responses to Ad infection, including DNA damage and interferon responses (3, 4). In the current study, we focus on the E4 open reading frame 3 (E4-ORF3) protein, which induces the relocalization of many cellular proteins into a unique and dynamic nuclear scaffold known as E4-ORF3 nuclear tracks (5). E4-ORF3 interacts with a specific isoform of the antiviral protein promyelocytic leukemia protein (PML/TRIM19) (6), disrupting PML nuclear bodies (NBs), a process that inhibits the intrinsic antiviral functions associated with these structures (7). E4-ORF3 also relocalizes proteins of the Mre11-Rad50-Nbs1 (MRN) DNA repair complex into E4-ORF3 nuclear tracks (8, 9). Redistribution of the MRN complex inhibits the activation of the DNA damage response (DDR), a complex cellular signaling cascade activated during Ad infection by the linear, double-stranded DNA genome (4). The Ad E4-ORF6/E1B-55K (55-kDa) protein complex also targets the MRN proteins for ubiquitin-mediated, proteasome-dependent degradation to inhibit their activity (10). If left unabated, the DDR leads to end-to-end concatenation of Ad genomes, a process that inhibits viral replication (4). Therefore, it is not surprising that Ad has evolved multiple mechanisms to counteract these responses.

Posttranslational modification of cellular proteins by the small ubiquitin-like (Ubl) modifier (SUMO) is critical for the regulation of transcription, replication, DNA repair, protein localization, protein stability, and protein-protein interactions (11). Growing evidence suggests that dysregulation of these modifications plays a critical role in a number of human disease processes. Interestingly, a number of DNA viruses manipulate host Ub and SUMO pathways, suggesting that these modifications play important roles in aspects of antiviral immunity (12, 13). In mammals, there are three ubiquitously expressed SUMO isoforms, SUMO1 to -3. SUMO2 and SUMO3 share 95% amino acid identity in precursor forms and 97% identity in mature forms, and they are often referred to as SUMO2/3, while SUMO1 and SUMO2/3 have only 50% identity and typically modify distinct substrates. Cellular substrates are mono-SUMOylated by SUMO1 but may be mono- or poly-SUMOylated by SUMO2/3. It is thought that SUMO2/3 modification is regulated more dynamically in response to various stimuli, such as heat shock, since the unconjugated SUMO2/3 population is larger than that of SUMO1 in mammalian cells (11).

We have previously shown that E4-ORF3 is required for the induction of SUMOylation of several track-associated proteins, including Mre11 and Nbs1 (14). E4-ORF3 is also necessary for reduced SUMOylation of PML protein during Ad infection (15). The consequence(s) of E4-ORF3-induced changes in SUMOylation of track proteins remains unknown. To better understand the process of E4-ORF3-induced SUMOylation and identify any additional SUMOylated targets of E4-ORF3, we recently employed a proteome-wide comparative analysis that revealed a diverse set of cellular substrates that were modified in response to E4-ORF3 expression. Among the list of substrates was the general transcription factor TFII-I, with the induction of Ubl modification at 37 independent sites and with a maximum 127-fold stimulation of Ubl modification (16).

TFII-I, encoded by the *GTF2I* gene, is a ubiquitously expressed transcription factor involved in the regulation of a wide array of biological processes, including growth factor signaling, cell cycle regulation and proliferation, and immune cell signaling (17). TFII-I can function as part of the basal transcription machinery and as a signal-inducible transcriptional activator or repressor (17). TFII-I was recently shown to be a negative regulator of the Ad5 intermediate-phase promoter, L4P, for which E4-ORF3 is an activator in reporter assays (18). Posttranslational modification of TFII-I is critical for its transcriptional activity at several promoters. TFII-I is tyrosine phosphorylated in response to various mitogenic and growth factor stimuli (19). Additionally, the activation of p53 following gamma irradiation leads to TFII-I ubiquitination and its proteasome-dependent degradation, demonstrating that ubiquitination of TFII-I is critical for cell cycle regulation in response to genotoxic stress (20). Interestingly, several reports, including ours, have demonstrated that TFII-I is SUMOylated under various conditions, including heat shock and viral infection (16, 21). SUMOylation of transcription factors is often correlated with transcriptional repression (11); however, the functional consequence of TFII-I SUMOylation in any context remains unknown.

In our current study, we sought to further characterize the regulation of TFII-I by E4-ORF3. We demonstrate that E4-ORF3 enhances both SUMO1 and SUMO3 modification of TFII-I. Interestingly, TFII-I turnover is enhanced by Ad infection and E4-ORF3 is sufficient for this to occur. Ad regulation of TFII-I does not appear to be a conserved process, since we show that it is specific to Ad5 E4-ORF3. E4-ORF3 induces TFII-I ubiquitination and promotes proteasome-dependent degradation of TFII-I. Finally, we show that E4-ORF3 augments the expression of the Ad5 delayed-early promoter, L4P, a previously identified target of TFII-I repression (18), in virus-infected cells. Taken together, our results demonstrate that E4-ORF3 regulates TFII-I by inducing both its SUMOylation and ubiquitination and show that E4-ORF3-induced degradation is likely a mechanism to counteract antiviral activities of TFII-I.

## RESULTS

### Wild-type Ad5 infection induces SUMO1 and SUMO3 modification of TFII-I.

**FIG 1:**
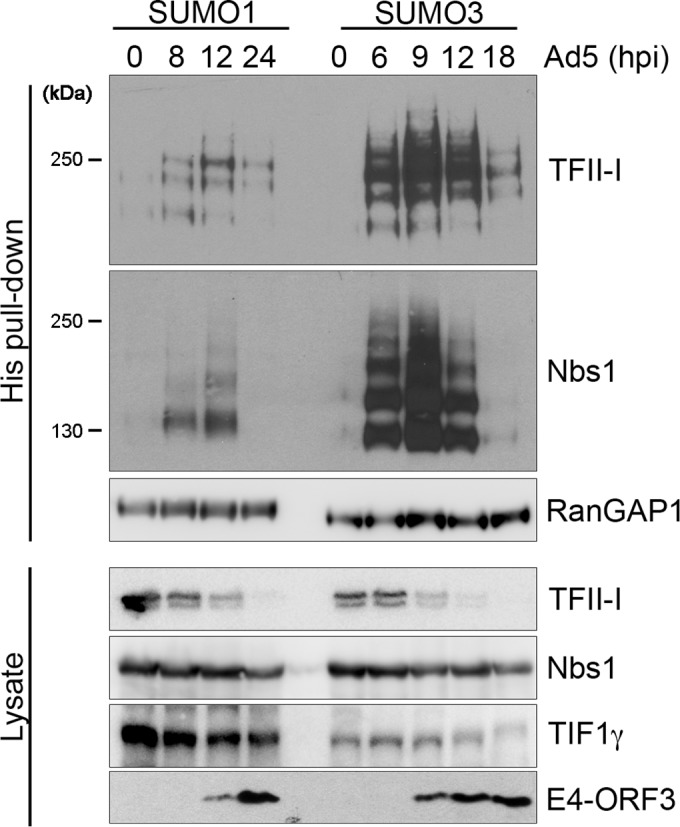
Paralog-specific SUMOylation of TFII-I during a time course of wild-type Ad5 infection. His6-SUMO1- and His6-SUMO3-expressing HeLa cells were infected with wild-type Ad5 and harvested for Ni-NTA affinity purification of SUMO-conjugated proteins at the indicated times (hours) postinfection (hpi). SUMO conjugates (His pull-down) were analyzed by Western blotting using antibodies directed against TFII-I, Nbs1, and RanGAP1. RanGAP1 was used as a control for SUMO capture, and Nbs1 served as a control for Ad-induced SUMOylation. Whole-cell lysates (Lysate) were analyzed by Western blotting for TFII-I, Nbs1, TIF1γ, and E4-ORF3.

Our previous studies demonstrated that TFII-I is modified by SUMO3 during Ad5 infection in an E4-ORF3-dependent manner (16). In our current study, we analyzed paralog-specific TFII-I SUMOylation during a time course of infection. HeLa cells stably expressing hexahistidine (His6)-tagged-SUMO1 or SUMO3 were infected with wild-type Ad5, and SUMO conjugates were isolated at different times postinfection and analyzed by Western blotting for TFII-I. SUMO3 modification of TFII-I was enhanced during Ad5 infection, and this modification peaked by 9 h postinfection and decreased thereafter. TFII-I modification by SUMO1 was also enhanced during Ad5 infection and exhibited similar, yet slightly delayed, kinetics compared with SUMO3 modification (Fig. 1, His pull-down). TFII-I SUMOylation correlated with a reduction in TFII-I protein levels (Fig. 1, Lysate). Ad-induced SUMO1 and SUMO3 modification of Nbs1, a previously described target (14), was observed, but there was no concomitant reduction in Nbs1 protein levels (Fig. 1, His pull-down and Lysate). In contrast, RanGAP1, a cellular protein that is constitutively modified by both SUMO1 and SUMO3, remained unchanged during infection. Previous studies demonstrated that Ad5 induces degradation of components of the MRN complex at late times postinfection (9). In our experiments, Nbs1 levels began to decrease by 18 h postinfection with wild-type Ad5 (Fig. 1, Lysate, Nbs1). In contrast, TFII-I levels were already decreased by 9 h postinfection and TFII-I was virtually undetectable at late times postinfection (Fig. 1, Lysate, TFII-I). Together, these data demonstrate that TFII-I modification by both SUMO1 and SUMO3 is enhanced during wild-type Ad5 infection and suggest that TFII-I may be targeted for degradation as a consequence.

### The E4-ORF3 protein is necessary and sufficient to reduce TFII-I protein levels.

**FIG 2:**
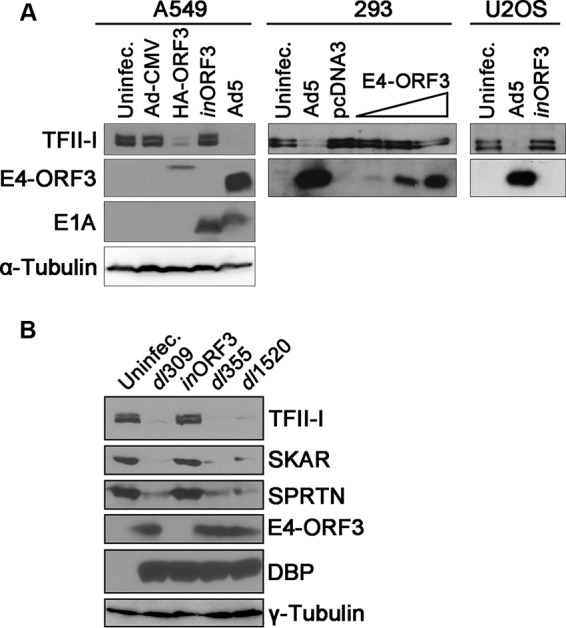
E4-ORF3 is necessary and sufficient to alter TFII-I protein levels. (A) A549, HEK 293, and U2OS cells were left uninfected or infected with wild-type Ad5, ΔE4-ORF3 virus (*in*ORF3), E1 replacement viruses containing only the CMV promoter (Ad-CMV) or expressing HA-tagged E4-ORF3 (HA-ORF3), or were transfected with pcDNA3 or increasing amounts of pcDNA3-E4-ORF3 (E4-ORF3). Cells were harvested at 18 h postinfection (A549) or 24 h postinfection (HEK 293 and U2OS cells) or at 24 h posttransfection (HEK 293 cells). Lysates were analyzed by Western blotting for TFII-I, E4-ORF3, E1A, and α-tubulin levels. (B) HeLa cells were left uninfected or infected with phenotypically wild-type Ad5 (*dl*309), ΔE4-ORF3 virus (*in*ORF3), ΔE4-ORF6 virus (*dl*355), or ΔE1B-55K virus (*dl*1520). Cells were harvested at 18 h postinfection, and whole-cell lysates were analyzed by Western blotting using the indicated antibodies. The Ad DNA binding protein (DBP) was analyzed as an infection control.

We further analyzed TFII-I protein levels during Ad5 infection. We infected A549, U2OS, and HEK 293 cells with wild-type Ad5 or an E4-ORF3-deficient virus (*in*ORF3) and examined TFII-I levels at 18 h (A549 cells) or 24 h (U2OS and HEK 293 cells) postinfection. TFII-I levels were decreased by wild-type infection but not by *in*ORF3 infection in all cell types examined (Fig. 2A); an E1-defective Ad vector that lacked E4-ORF3 expression and contained the cytomegalovirus promoter (Ad-CMV) failed to reduce TFII-I levels in A549 cells, and this ability was restored by incorporating an E4-ORF3 transgene into the vector (HA-E4-ORF3). Both results demonstrate that E4-ORF3 is required for the observed reduction in TFII-I protein levels. We next examined the ability of ectopically expressed E4-ORF3 to induce changes in TFII-I levels and found that transient expression of E4-ORF3 was sufficient to downregulate TFII-I (Fig. 2A, HEK 293 cells).

It is well established that Ad5 infection promotes the degradation of several cellular DNA repair proteins (10). Previous studies attributed this to the E4-ORF6 and E1B-55K proteins, but more recently, E4-ORF3 was also shown to mediate degradation of TIF1γ/TRIM33 independently of any other viral factors (22). Therefore, we examined the ability of Ads containing mutations in E4-ORF6 or E1B-55K to induce changes in TFII-I protein levels at late times during infection. The levels of TFII-I in cells infected with an E4-ORF6 mutant (*dl*355) or an E1B-55K mutant (*dl*1520) were greatly reduced at 18 h postinfection, similar to the levels observed in wild-type virus-infected cells (*dl*309), demonstrating that loss of TFII-I occurs independently of E4-ORF6 and E1B-55K (Fig. 2B). Our previous proteomic studies analyzing global E4-ORF3-induced Ubl modifications revealed several novel targets in addition to TFII-I (16). We analyzed the levels of two of these proteins, SKAR (POLDIP3) and SPRTN (C1orf124), to determine whether their expression levels were also affected by E4-ORF3. Consistent with the effect of E4-ORF3 on TFII-I, the levels of both SKAR and SPRTN in the presence of E4-ORF3 were significantly reduced by 18 h postinfection with wild-type Ad5 (*dl*309) but not with *in*ORF3; this effect was independent of E4-ORF6 or E1B-55K (Fig. 2B). Together, these results show that the loss of TFII-I during Ad5 infection is not cell-type specific and that E4-ORF3 is necessary and sufficient to cause a decrease in TFII-I levels. In addition, E4-ORF3 expression also results in decreases in the levels of other cellular proteins whose modification is regulated by E4-ORF3.

### E4-ORF3-mediated relocalization of TFII-I is necessary for its SUMOylation and degradation.

**FIG 3:**
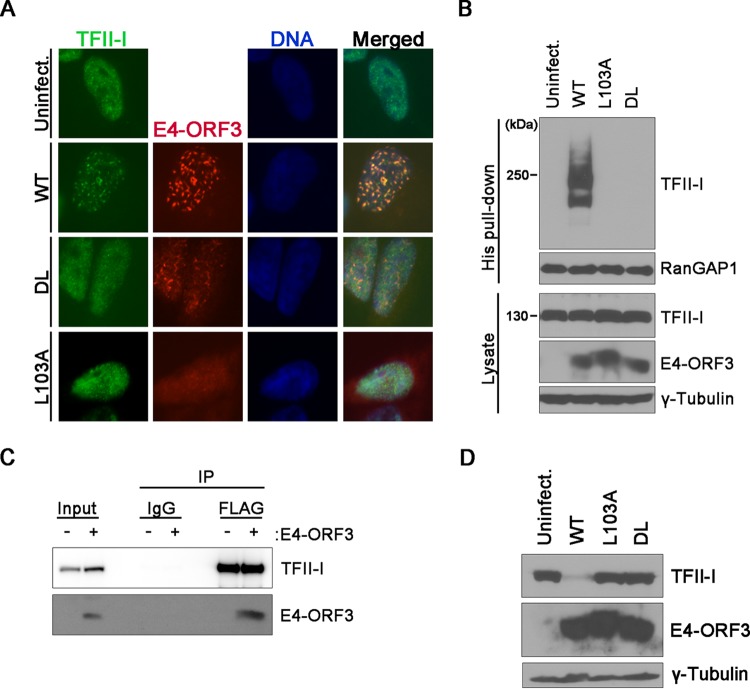
E4-ORF3-mediated relocalization of TFII-I is required for its SUMOylation and reduction in protein levels. (A) HeLa cells were left uninfected or infected with viruses expressing wild-type E4-ORF3 (WT) or one of two E4-ORF3 mutants, D_105_A/L_106_A (DL) or L_103_A (L_103_A). Cells were fixed at 7 h postinfection and immunostained using antibodies against TFII-I and E4-ORF3. Nuclei were visualized by 4[prime],6-diamidino-2-phenylindole (DAPI) staining (DNA). Merged images are shown in the far-right column. (B) His6-SUMO3-expressing HeLa cells were infected as described in the legend to panel A. Cell extracts were prepared at 7 h postinfection for Ni affinity purification and analyzed as described in the legend to Fig. 1. (C) HeLa cells were transfected with pCMV-3Tag-TFII-I and infected the following day with Ad-CMV expressing wild-type HA-E4-ORF3. Cell extracts were prepared at 12 h postinfection, and TFII-I was immunoprecipitated using anti-FLAG antibody (FLAG) or an isotype-matched IgG control (IgG). Immunoprecipitated proteins were separated by SDS-PAGE and analyzed by Western blotting for TFII-I (anti-FLAG antibody) and E4-ORF3 (anti-HA antibody). (D) HeLa cells were infected as described above and harvested at 18 h postinfection. Whole-cell lysates were analyzed by Western blotting for the indicated proteins.

We next examined E4-ORF3-mediated SUMOylation and localization of TFII-I. Due to compensatory roles of E4-ORF3 and E4-ORF6 in targeting certain host antiviral responses, we analyzed E4-ORF3-specific effects on TFII-I using two well-characterized E4-ORF3 point mutants which were expressed in the background of an E4-ORF6-deficient virus (*dl*355). The E4-ORF3 point mutant, with a mutation of L to A at position 103 (*dl*355pmL_103_A), is defective in all E4-ORF3-associated functions (23). The E4-ORF3 double point mutant, with mutations of D to A at position 105 and L to A at position 106 (*dl*355pmD_105_A/L_106_A), is defective in the relocalization of some target proteins, such as the MRN complex, but effectively directs the relocalization of others, such as SUMO and PML (8, 14, 24). Infection with a virus that expresses wild-type E4-ORF3 (*dl*355) resulted in redistribution of TFII-I to nuclear tracks (Fig. 3A, WT). In contrast, the TFII-I localization remained unchanged in cells expressing the nonfunctional E4-ORF3 L_103_A mutant protein or the D_105_A/L_106_A mutant protein (Fig. 3A, L103A, DL). These observations are consistent with the inability of these E4-ORF3 mutants to induce SUMOylation of TFII-I (Fig. 3B). Since TFII-I colocalized with E4-ORF3, we next investigated whether TFII-I could interact with E4-ORF3. Coimmunoprecipitation analysis showed that TFII-I associates with E4-ORF3 *in vivo* (Fig. 3C). Finally, we analyzed the ability of E4-ORF3 to reduce the levels of TFII-I protein during infection and found that TFII-I protein levels were not reduced in cells expressing either the L_103_A or the D_105_A/L_106_A E4-ORF3 mutant protein (Fig. 3D). We conclude that relocalization of TFII-I to E4-ORF3 nuclear tracks directly correlates with TFII-I SUMOylation and a reduction in TFII-I protein levels.

### The effect on TFII-I protein levels is Ad serotype specific.

**FIG 4:**
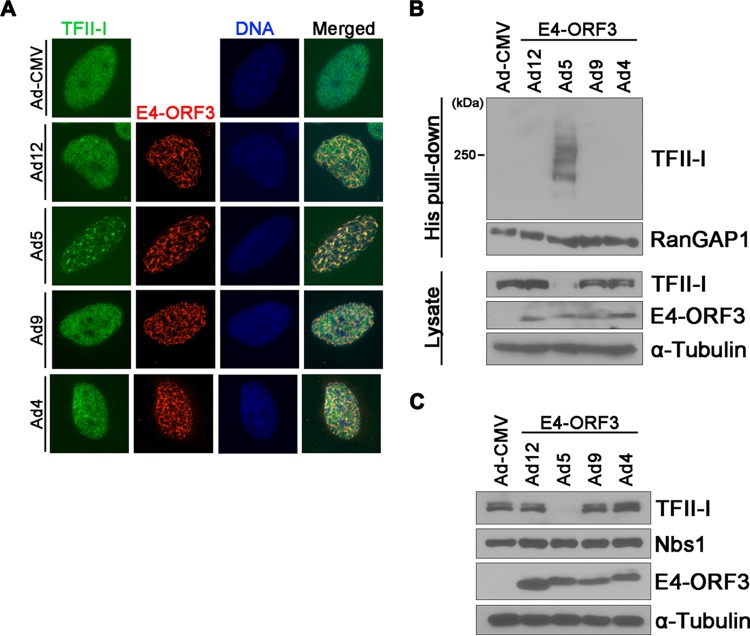
The effect on TFII-I protein levels is Ad serotype specific. (A) TFII-I subcellular localization was examined in HeLa cells infected with Ad-CMV viruses that express HA-E4-ORF3 from Ad12, Ad5, Ad9, or Ad4. Cells were fixed at 7 h postinfection and immunostained for TFII-I and E4-ORF3 (anti-HA antibody). Nuclei were visualized using DAPI staining (DNA). Merged images are shown in the far-right column. (B) His6-SUMO3-expressing HeLa cells were infected as described in the legend to panel A. Cell extracts were prepared at 7 h postinfection for Ni affinity purification and analyzed as described in the legend to Fig. 1. (C) HeLa cells were infected as described in the legend to panel A. Whole-cell extracts were prepared at 18 h postinfection and analyzed by Western blotting using the indicated antibodies.

Our previous studies showed that Ad5 E4-ORF3, but not E4-ORF3 from divergent human Ad species, induced SUMOylation of MRN proteins (14). Previous studies also showed that Ad5 (species C) E4-ORF3 is able to induce relocalization of the MRN complex, while Ad4 and Ad12 (species E and A) E4-ORF3 cannot (25). Some E4-ORF3 processes are conserved among divergent Ads, including the ability of E4-ORF3 to induce relocalization of PML NBs and SUMO proteins (14, 25). We sought to determine whether targeting of TFII-I is conserved across the different Ad species A, C, D, and E (serotypes 12, 5, 9, and 4, respectively). Cells were infected with recombinant Ad vectors expressing different hemagglutinin (HA)-tagged E4-ORF3 proteins or Ad-CMV. As expected, TFII-I colocalized with Ad5 E4-ORF3; however, TFII-I localization remained unaffected by E4-ORF3 from Ad12, Ad9, and Ad4, instead displaying diffuse nuclear localization similar to that in the control cells infected with Ad-CMV (Fig. 4A). We next analyzed the ability of E4-ORF3 proteins from different Ad serotypes to induce TFII-I SUMOylation and found that only Ad5 E4-ORF3 was able to enhance SUMO3 conjugation to TFII-I (Fig. 4B). To further characterize the functional relationship between E4-ORF3 and TFII-I, we evaluated TFII-I protein levels in the presence of divergent E4-ORF3 proteins. A reduction in TFII-I protein levels was observed only in the presence of Ad5 E4-ORF3 and not with Ad12, Ad9 or Ad4 E4-ORF3 proteins (Fig. 4B and C). These data demonstrate that regulation of TFII-I SUMOylation and protein levels by E4-ORF3 is not conserved across human Ads and is specific to Ad5 E4-ORF3 from species C. These results also strengthen the direct correlation between Ad5 E4-ORF3-induced relocalization and SUMOylation of TFII-I and a significant reduction in TFII-I protein levels.

### E4-ORF3-mediated regulation of TFII-I expression levels is posttranscriptional.

**FIG 5:**
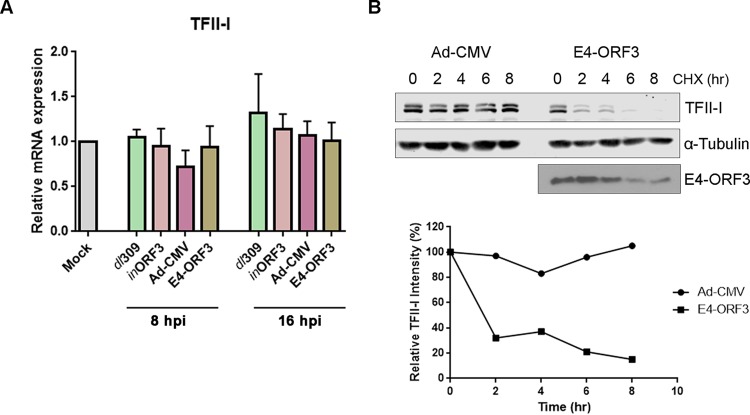
E4-ORF3-mediated modulation of TFII-I protein levels is posttranscriptional. (A) HeLa cells were left uninfected (Mock) or infected with wild-type Ad5 (*dl*309), ΔE4-ORF3 virus (*in*ORF3), Ad-CMV, or Ad-CMV-HA-ORF3-WT. Total RNA was isolated at 8 and 16 h postinfection, and TFII-I mRNA levels were quantified by RT-qPCR and normalized to cellular glyceraldehyde-3-phosphate dehydrogenase (GAPDH). The results shown represent the mean values ± standard deviations (SD); *n* = 3. (B) HeLa cells were infected with Ad-CMV or Ad-CMV-HA-E4-ORF3 and treated with cycloheximide beginning at 6 h postinfection. Whole-cell lysates were prepared at the indicated time points following the addition of cycloheximide and analyzed by Western blotting for TFII-I and E4-ORF3. The Western blots were quantified, and relative TFII-I intensities plotted against time post-cycloheximide addition. α-Tubulin served as a loading control.

E4-ORF3 alters the localization of many cellular proteins important for transcriptional regulation; therefore, it is not surprising that E4-ORF3 regulates cellular gene expression. Previous microarray experiments demonstrated that E4-ORF3 regulates a number of different cellular genes but not *GTF2I* (TFII-I) (26, 27). In order to rule out any regulatory effects E4-ORF3 may have on TFII-I gene expression, we analyzed TFII-I transcript levels in infected HeLa cells in the presence and absence of E4-ORF3 by reverse transcription-quantitative PCR (RT-qPCR). E4-ORF3 expression, whether from the E4 region or from the CMV promoter in an Ad vector, had no effect on TFII-I transcript levels either at early (8 h) or late (16 h) times postinfection (Fig. 5A). These results suggested that E4-ORF3 may affect TFII-I protein levels by regulating the half-life of TFII-I protein. To examine this possibility, HeLa cells were infected with Ad-CMV or Ad-CMV-HA-E4-ORF3 and subsequently treated with cycloheximide for different lengths of time to inhibit protein translation. In the presence of E4-ORF3, TFII-I turnover was drastically enhanced compared with its turnover in the control infection (Fig. 5B), demonstrating that E4-ORF3 stimulates TFII-I degradation.

### E4-ORF3 stimulates TFII-I ubiquitination and targets TFII-I for proteasome-dependent degradation.

**FIG 6:**
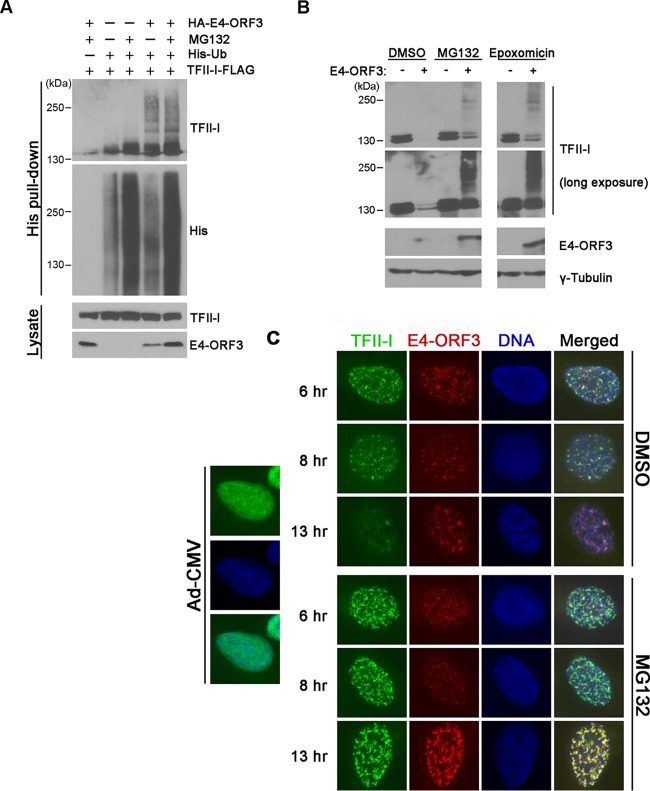
E4-ORF3 induces ubiquitination and proteasome-dependent degradation of TFII-I. (A) HeLa cells expressing His_12_-Ubiquitin (His-Ub) (third row, +) and TFII-I-FLAG (bottom row, +) were infected with Ad-CMV (top row, −) or Ad-CMV-HA-E4-ORF3 (top row, +). At 1 h postinfection, cells were treated with DMSO (second row, −) or 10 µM MG132 (second row, +) for 12 h. His-Ub conjugates were enriched from whole-cell extracts using Ni affinity purification and analyzed by Western blotting. TFII-I and ubiquitinated proteins were detected using anti-FLAG (TFII-I) and anti-His antibodies, respectively. TFII-I and E4-ORF3 protein levels were analyzed from whole-cell lysates (Lysate) using anti-FLAG and anti-HA antibodies, respectively. (B) HeLa cells were infected with Ad-CMV (−) or Ad-CMV-HA-E4-ORF3 (+). At 1 h postinfection, cells were treated with DMSO, 10 µM MG132, or 2 µM epoxomicin for 12 h. Whole-cell extracts were prepared and analyzed by Western blotting for TFII-I and E4-ORF3 protein levels; γ-tubulin was used as a loading control. (C) HeLa cells were infected with Ad-CMV (left) or Ad-CMV-HA-E4-ORF3 (right). At 1 h postinfection, cells were treated with DMSO or 10 µM MG132. Cells were fixed at the indicated times postinfection and immunostained for TFII-I and E4-ORF3. Nuclei were visualized by DAPI (DNA). Merged images are shown in the far-right column. The fluorescein isothiocyanate (FITC) channel exposure time was identical for each image; shorter exposure times were used at late times postinfection with E4-ORF3 images (tetramethyl rhodamine isothiocyanate [TRITC] channel) due to high levels of protein expression.

Ubiquitin modification of target proteins is often associated with the regulation of protein stability. We therefore assessed the effect of E4-ORF3 on TFII-I ubiquitination by infecting HeLa cells expressing His_12_-tagged ubiquitin with Ad-CMV or Ad-CMV-HA-E4-ORF3 in the presence or absence of a proteasome inhibitor. Cells were harvested 11 h postinfection, and ubiquitin-conjugated proteins were affinity purified from total cell lysates. Interestingly, TFII-I ubiquitination was enhanced in the presence of E4-ORF3 compared with the results for control-infected cells (Fig. 6A, His pulldown, TFII-I, 2nd and 4th lanes). Likewise, ubiquitin modification of TFII-I was further enhanced by treatment with the proteasome inhibitor MG132 (Fig. 6A, His pulldown, TFII-I, 5th lane), which, as expected, stabilized the pool of ubiquitin-modified substrates (Fig. 6A, His pulldown, His, lanes 3 and 5). These results indicate that E4-ORF3 induces ubiquitination of TFII-I and suggest that the turnover of TFII-I in response to E4-ORF3 may be mediated by the proteasome.

We sought to determine the mechanism of TFII-I degradation. HeLa cells were infected with Ad-CMV (Fig. 6B, −) or Ad-CMV-HA-E4-ORF3 (Fig. 6B, +) and then treated with dimethyl sulfoxide (DMSO; vehicle) or a proteasome inhibitor, MG132 or epoxomicin, from 1 to 12 h postinfection. As expected, TFII-I was significantly reduced following E4-ORF3 expression in control cells (Fig. 6B, DMSO). TFII-I protein levels were partially restored by MG132 or epoxomicin treatment and higher-molecular-weight TFII-I species were evident, consistent with the stabilization of TFII-I-ubiquitin conjugates (Fig. 6B). Interestingly, E4-ORF3 protein levels were also increased in the presence of both proteasome inhibitors, suggesting that E4-ORF3 itself may be subject to proteasome-dependent degradation (Fig. 6B). Finally, we compared TFII-I localization and expression levels by immunofluorescence in the presence or absence of MG132 during a time course of infection using the Ad-CMV-HA-E4-ORF3 expression vector. Supporting what was observed by Western blotting, the TFII-I protein levels were noticeably increased in the presence of the drug, and the protein continued to be associated with E4-ORF3 nuclear tracks at late times postinfection (Fig. 6C). Taken together, our results demonstrate that E4-ORF3 regulates TFII-I by inducing ubiquitination and proteasome-dependent degradation.

### E4-ORF3 augments the expression of the Ad5 intermediate promoter L4P, a target of TFII-I repression, in virus-infected cells.

**FIG 7:**
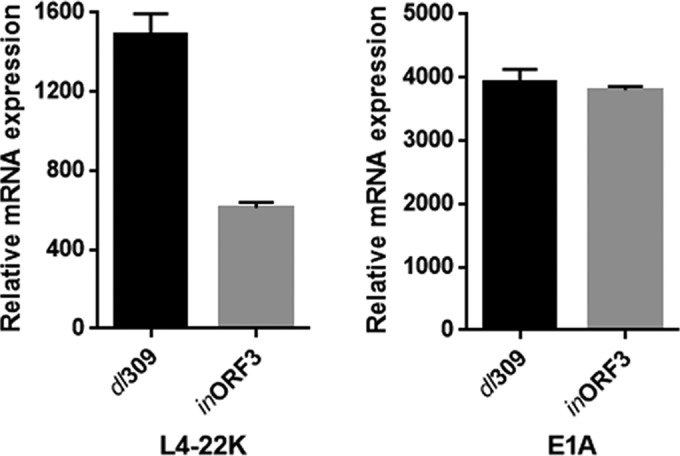
E4-ORF3 stimulates L4P in virus-infected cells. HeLa cells were infected with *dl*309 or *in*ORF3, and total cellular RNA was isolated at 9.5 h postinfection. E1A and L4-22K mRNA levels were quantified by RT-qPCR and normalized to endogenous GAPDH mRNA levels. The results shown represent the mean values ± SD; *n* = 3.

We recently demonstrated that TFII-I negatively regulates the Ad5 intermediate promoter, L4P (18). L4P activity is regulated by multiple factors, including the Ad5 proteins E1A, E4-ORF3, and IVa2, as well as by a cellular stress response via p53, and the effect of E4-ORF3 required an inhibitory TFII-I binding site in the promoter (18, 28, 29). We therefore examined whether E4-ORF3 regulates L4P during viral infection. We infected HeLa cells with wild-type Ad5 (*dl*309) and an E4-ORF3 mutant virus (*in*ORF3) and quantified L4P activity by RT-qPCR at 9.5 h postinfection, a time when L4P-derived mRNAs are evident (18); E1A mRNAs were quantified as an internal control for comparable infectivity. E4-ORF3 stimulated L4 mRNA levels by ~2.5-fold during virus infection (Fig. 7), an effect that is comparable in scale to that previously reported using transient expression assays (28, 29). E1A transcript levels were equivalent in infections with the two viruses. We conclude that E4-ORF3 stimulates the expression of the Ad5 L4P promoter in virus-infected cells, consistent with its ability to induce TFII-I degradation at this time postinfection.

## DISCUSSION

We previously reported that Ad5 E4-ORF3 induces relocalization and SUMOylation of TFII-I (16). In our current studies, we identify a new mechanism by which E4-ORF3 modulates TFII-I, by regulating its stability via inducing TFII-I ubiquitination and proteasomal degradation during infection. We show that TFII-I is targeted by E4-ORF3 for modification with both SUMO1 and SUMO3 and that these modifications are reduced at later times during the course of infection. It appears that the pool of SUMO-modified TFII-I decreased due to protein turnover rather than SUMO deconjugation, given the marked reduction in the total pool of TFII-I at late times after infection. Given that the majority of Ad-induced proteolysis occurs via the E4-ORF6 and E1B-55K proteins (10), we looked for the requirement of these viral factors in the loss of TFII-I. We found that neither E4-ORF6 nor E1B-55K affect TFII-I levels and further demonstrated that E4-ORF3 is both necessary and sufficient to modulate TFII-I. E4-ORF3 targets the antiviral protein TIF1γ for proteasomal degradation, independent of other viral proteins, and this is a conserved function of the E4-ORF3 proteins of multiple Ad serotypes (22). In contrast, we found that E4-ORF3-induced degradation of TFII-I was not conserved among Ad serotypes and that modulation of TFII-I by E4-ORF3 was specific to Ad5. This serotype specificity is consistent with studies of E4-ORF3 targeting of MRN complex proteins, where only Ad5 E4-ORF3 relocalizes and induces SUMOylation of components of this complex (25). Ad5 E4-ORF3, however, does not induce the degradation of MRN complex proteins (9). These results underscore the complex mechanisms that the E4-ORF3 proteins of different human Ads have evolved to regulate the activity of multiple cellular proteins.

Recently, we found that TFII-I negatively regulates the Ad5 intermediate L4 promoter (L4P) through its binding to an initiator element (Inr) within L4P and, thus, has antiviral activity. We demonstrated that in transient expression assays, TFII-I’s repression of L4P was relieved by E4-ORF3 (18). Additionally, mutation of the Inr element within L4P increased the basal activity of the promoter, correlating with the loss of TFII-I binding and demonstrating that TFII-I functions to repress L4P activity. However, the mechanism of E4-ORF3 regulation of TFII-I during infection remained unknown. Here, we demonstrated that E4-ORF3 stimulates L4 expression during the early phase of infection with wild-type Ad5. We attribute this function of E4-ORF3 to the degradation of TFII-I and, thereby, relief of TFII-I-mediated L4P repression. The fact that E4-ORF3 only increased L4P expression by ~2.5-fold in these assays may reflect the redundant nature of functional elements within Ad promoters and the fact that multiple Ad gene products stimulate L4P activity (18, 28). Since L4P directs the production of crucial protein activators of viral late gene expression, even modestly increased L4P activity will be a selective advantage for the virus. These results correlate well with the two- to threefold reduction in virus yield observed with E4-ORF3 mutants (30).

E4-ORF3 is a small, highly conserved protein whose oligomerization properties are conserved across Ad serotypes from all human Ad species, and studies indicate that this function of E4-ORF3 is required for its interaction with cellular proteins (23, 31). E4-ORF3 targets a number of different cellular complexes, including PML NBs and MRN, and studies indicate that individual interactions with its substrates are mediated through unique binding interfaces within the E4-ORF3 protein (31). Consistent with previous studies from our laboratory (8), recent evidence demonstrates that the interaction of E4-ORF3 with the MRN complex is mediated by individual C-terminal residues; specifically, V_101_ to D_105_ (31). Because only Ad5 E4-ORF3 targets TFII-I and previous studies demonstrated that the same is true for E4-ORF3 targeting of the MRN complex (25), we compared the ability of two previously characterized E4-ORF3 mutants, one globally deficient (L_103_A mutant) and the other selectively deficient (D_105_A/L_106_A mutant) in MRN reorganization (8, 24), to modulate TFII-I. As expected, targeting of TFII-I by E4-ORF3 requires its ability to form tracks, since the L_103_A mutant lacking this ability did not induce changes in TFII-I localization, SUMOylation, or stability. Likewise, analysis of cells infected with the D_105_A/L_106_A mutant showed that these residues are also important for the ability of E4-ORF3 to modulate TFII-I.

Our previous results suggest that recruitment of cellular proteins into E4-ORF3 tracks is required for their SUMOylation (14). These results suggest that relocalization into E4-ORF3 tracks precedes E4-ORF3-induced SUMOylation; however, additional experiments are needed to delineate whether E4-ORF3 first recruits TFII-I and other proteins into tracks prior to inducing their SUMOylation or whether E4-ORF3 usurps the cellular SUMO machinery as a mechanism to recruit proteins into tracks. It is possible that one or more SUMO E3 ligases are recruited into tracks to catalyze SUMOylation of track-associated substrates. E4-ORF3 recruits a number of different SUMO ligases to tracks, including several members of the TRIM family, PML (TRIM19) and TIF1γ (TRIM33), as well as PIAS3 (5, 27, 32). Although it lacks the canonical RING domain common to cellular SUMO E3 ligase enzymes, it is possible that E4-ORF3 itself functions as a SUMO ligase or otherwise promotes protein SUMOylation. Future studies will be aimed at determining how E4-ORF3 functions to stimulate cellular protein SUMOylation.

One of the questions that remains is specifically how E4-ORF3 regulates cellular protein stability. Our results are consistent with the idea that SUMOylation is involved in this process, but we also showed that elevated ubiquitination is a direct cause of proteasomal targeting of TFII-I. Previous studies explored the possibility that E4-ORF3 utilizes a cullin-based Ub ligase complex to facilitate the degradation of TIF1γ, a target of E4-ORF3-induced proteolysis (22). The cullin 2/5 proteins are key components of the E4-ORF6/E1B-55K-Ub ligase complexes of different Ad serotypes and provide a scaffold to mediate ubiquitin-dependent degradation of numerous antiviral host proteins during infection (33, 34). However, neither Cul2 nor Cul5 mediated E4-ORF3-induced degradation of TIF1γ (22); whether E4-ORF3 utilizes these particular Cul-Ub ligase complexes to facilitate TFII-I degradation remains to be explored, although this seems unlikely given that the Cul complexes have only been associated thus far with E4-ORF6/E1B-55K. Another intriguing possibility is that E4-ORF3 induces SUMOylation of target proteins as a molecular signal to regulate protein degradation during infection. Growing evidence suggests that SUMOylation plays a critical role in protein stability, serving as a signal for protein ubiquitination by a recently identified class of enzymes called SUMO-targeted Ub ligases, or STUbLs (35). To date, two STUbLs have been identified in mammalian cells, RNF4 and RNF111, which contain multiple SUMO-interacting motifs (SIMs) and have high affinity for poly-SUMOylated proteins (36, 37). RNF4 has been shown to be critical for efficient DNA repair processes, functioning to regulate the levels of SUMOylated cellular repair proteins (35), making it an attractive candidate in the context of Ad infection. Interestingly, the herpesvirus protein ICP0, which shares its PML NB-targeting capability with that of E4-ORF3, functions as a STUbL that preferentially ubiquitinates poly-SUMOylated PML during herpesvirus infection as a mechanism to inhibit the antiviral activities of PML (38). Our data demonstrate that E4-ORF3 is sufficient to induce TFII-I degradation; however, whether E4-ORF3 itself exhibits STUbL-like properties or whether E4-ORF3 recruits cellular STUbLs in order to facilitate degradation of SUMOylated proteins, such as TFII-I, remains to be explored. We noted that the levels of two other cellular proteins, SKAR and SPRTN, whose modification is induced by E4-ORF3 (16), also were greatly reduced by Ad5 infection, indicating that E4-ORF3 may target numerous substrates for SUMO-mediated, ubiquitin-dependent degradation. A reduction in POLDIP3/SKAR protein expression following wild-type Ad5 infection was previously described based on a proteome and transcriptome screen, although the basis of this observation was not pursued (39).

Finally, Western blot and immunofluorescence results indicate that the E4-ORF3 protein itself may be degraded via the proteasome, since E4-ORF3 levels were augmented by MG132 and epoxomicin treatment (Fig. 6B and C). Perhaps E4-ORF3’s interaction with substrates targeted for proteasomal degradation mediates this process. In conclusion, we have shown that E4-ORF3 regulates both TFII-I SUMOylation and ubiquitination. We have also demonstrated that E4-ORF3 targets TFII-I for proteasomal degradation, a function that correlates with its effects on SUMOylation and ubiquitination. Since E4-ORF3 was previously documented to affect TFII-I localization (16), we believe that Ad has evolved several redundant mechanisms to counteract the intrinsic antiviral properties of TFII-I. One effect of this targeting is to increase gene expression from Ad5 L4P. These findings suggest that the regulation of protein ubiquitination and degradation by E4-ORF3 is an underappreciated mechanism that Ad exploits to modulate its replicative environment to maximize the success of infection.

## MATERIALS AND METHODS

### Viruses and infections.

The viruses used in these studies, *dl*309 (phenotypically wild-type Ad5), wild-type Ad5, *in*ORF3 (ΔE4-ORF3), *dl*355 (ΔE4-ORF6), *dl*1520 (ΔE1B-55K), *dl*355pmL_103_A, and *dl*355pmD_105_A/L_106_A (ΔE4-ORF6 background with point mutations in E4-ORF3), and hemagglutinin (HA)-tagged E4-ORF3 proteins from Ad4, Ad5, Ad9, and Ad12 expressed using Ad5 E1 replacement viruses under the control of a CMV promoter were previously described (8, 14, 30). Viral infections were performed at multiplicities of infection of 200 to 600 virus particles/cell with wild-type Ad5 and mutant viruses and 500 to 2,000 virus particles/cell with Ad-CMV vectors in order to normalize E4-ORF3 expression levels. HEK 293 and U2OS cells were infected at 10 PFU/cell and 30 PFU/cell, respectively.

### *In vivo* SUMOylation assay.

HeLa cells or His6-SUMO1- or His6-SUMO3-expressing HeLa cells were left uninfected or infected with Ads as indicated in the text, and cells were harvested at 7 h postinfection. SUMO conjugates were prepared as previously described and analyzed by Western blotting (40).

### TFII-I protein half-life.

HeLa cells were infected with Ad-CMV or Ad-CMV-HA-E4-ORF3 at 1,000 particles/cell. At 6 h postinfection, cycloheximide (CHX) was added to the medium to a final concentration of 60 µg/ml, and cells were harvested at the time points indicated in the text. Equal amounts of lysates were analyzed by Western blotting. TFII-I levels were quantified using Image Studio software (LI-COR Biosciences).

### Ubiquitination assay.

HeLa cells were transfected with His_12_-tagged Ub and FLAG-tagged TFII-I expression vectors using polyethyleneimine (PEI). After 20 h, cells were infected with viruses as indicated in the text; 10 µM MG132 (Calbiochem) was added at 1 h postinfection, and the cells were incubated for 11 h. Cells were harvested, resuspended in buffer A (6 M guanidine-HCl, 0.1 M sodium phosphate, and 10 mM imidazole, pH 8.0), and lysed by sonication. After 3 h of incubation with Ni-nitrilotriacetic acid (NTA) agarose beads (Qiagen) at room temperature, the beads were washed once with buffer A, once with a 1:3 mixture of buffer A and buffer TI (20 mM imidazole, 0.2% Triton X-100, and 25 mM Tris-Cl, pH 6.8), and 3 times with buffer TI. The beads were boiled in 2× SDS sample buffer containing 200 mM imidazole, and eluates were analyzed by Western blotting.

Additional materials and methods may be found in Text S1 in the supplemental material.

## SUPPLEMENTAL MATERIAL

Text S1  Additional materials and methods. Download Text S1, PDF file, 0.1 MB
