# Supplementary material for: The Adenovirus E4-ORF3 Protein Stimulates SUMOylation of General Transcription Factor TFII-I to Direct Proteasomal Degradation
Source: mBio. 2016 Jan 26;7(1):e02184-15. doi: 10.1128/mBio.02184-15 (PMC4742714; doi:10.1128/mBio.02184-15)
Supplement: Text S1 — Additional materials and methods. Download [file mbo001162644s1.pdf]

SUPPLEMENTAL MATERIALS AND METHODS

Plasmids and cells. Expression plasmid pCMV-3Tag-TFII-I was constructed by PCR amplification of the TFII-I coding sequence of pEBB-II-I-GFP gamma isoform (a gift from Ananda Roy, Addgene #22148, previously described in [1]); PCR products were then cloned into pCMV-3Tag-3A (Agilent Technologies) to generate a C-terminally FLAG-tagged TFII-I. A His_{12}-tagged ubiquitin expression plasmid was provided by Dr. Dafna Bar-Sagi (New York University, New York, NY). Experiments were performed using HeLa, A549, 293, or U2OS (ATCC) cells maintained in Dulbecco's modified Eagle's medium (DMEM) containing 10% fetal bovine serum (HyClone Laboratories). HeLa cell lines stably expressing either His_6-tagged SUMO1 or SUMO3 [2] were generously provided by Dr. Ronald Hay (University of Dundee, Dundee, Scotland, UK) and maintained in DMEM supplemented with 10% fetal bovine serum and 1 μg/ml of puromycin.

Antibodies. Anti-TFII-I rabbit polyclonal antibodies were purchased from Cell Signaling Technologies and Santa Cruz Biotechnology, and an anti-SKAR rabbit monoclonal antibody was purchased from Cell Signaling Technologies. The anti-C1orf124 (SPRTN) mouse monoclonal antibody was kindly provided by Dr. Yuichi Machida (Mayo Clinic, Rochester, MN). The anti-HA (5B1D10) mouse monoclonal antibody was purchased from Life Technologies and the anti-HA rabbit polyclonal antibodies were purchased from Rockland Immunochemicals and Abcam. The anti-E1A mouse monoclonal (M73), anti-His_6 polyclonal, and anti-RanGAP1 mouse monoclonal antibodies were purchased from Santa Cruz Biotechnology. The anti-E4-ORF3 (6A11) rat monoclonal antibody was provided by Dr. Thomas Dobner (Heinrich-Pette Institute, Hamburg, Germany) and the anti-DBP (B6-8) mouse monoclonal antibody was provided by Dr. Arnold Levine (Princeton University, Princeton, NJ). The anti-α-Tubulin mouse monoclonal, anti-γ-Tubulin rabbit polyclonal, and anti-FLAG (M2) mouse monoclonal antibodies were purchased from Sigma-Aldrich. The anti-Nbs1 mouse monoclonal antibody was purchased from GeneTex and the anti-TIF1γ rabbit polyclonal antibody was previously described [3].

Immunofluorescence. HeLa cells were grown on glass coverslips in a 24-well plate and were uninfected or infected with the indicated Ads, as previously described [4]. At the indicated times post-infection, cells were fixed in methanol, blocked in PBS containing 10% goat serum, and proteins were detected using the indicated primary antibodies and fluorescein isothiocyanate- (FITC) or tetramethyl rhodamine isothiocyanate- (TRITC) conjugated secondary antibodies (Millipore). Cells were imaged using an Axiovert 200M digital deconvolution microscope (Zeiss) and images were analyzed using Axiovision software.

Immunoprecipitation. The pCMV-3Tag-TFII-I plasmid was transfected into HeLa cells using polyethyleneimine (PEI, Polysciences). After 20 hours incubation, cells were infected with Ad-CMV or Ad-CMV-HA-E4-ORF3. Twelve hours post-infection, cells were resuspended in lysis buffer (20 mM Tris-Cl pH 7.5, 50 mM NaCl, 1 mM EDTA, 0.5% NP-40, and the following inhibitors: 2 mM N-ethylmaleimide (NEM), 5 mM iodoacetamide (IAA), 1 mM PMSF, 1 mM sodium fluoride (NaF), 5 mM sodium orthovanadate, 1 mM benzamidine HCl, 2 μg/mL aprotinin, 2 μg/mL leupeptin, 1 μg/mL pepstatin A) and lysates were sonicated. After centrifugation, lysates were precleared using protein G-agarose beads (Roche) for one hour and incubated with anti-FLAG (M2, Sigma Aldrich) antibody overnight followed by incubation with protein G-agarose for 3 hours. The beads were washed five times with lysis buffer and retained proteins eluted in SDS sample buffer and analyzed by Western blotting.

RT-qPCR. For measurement of TFII-I mRNA levels, HeLa cells were infected with *dl*309 or *in*ORF3 at 200 particles/cell, or Ad-CMV or Ad-CMV-HA-E4-ORF3 at 500 particles/cell, and total RNA was isolated at 8 and 16 hours post-infection using a QIAGEN RNeasy kit**.** For measurement of E1A and L4-22K mRNA levels, HeLa cells were infected with *dl*309 or *in*ORF3 at 200 particles/cell and total RNA was isolated at 9.5 hours post-infection. Equal amounts of RNA from each sample (1 ug) were used for synthesis of first-strand cDNA using SuperScript II reverse transcriptase (Life Technologies) and quantified by qPCR using primer pairs specific for cellular TFII-I and GAPDH mRNAs, or viral E1A and L4-22K mRNAs, with DyNAmo HS SYBR

Green qPCR Kit (Thermo). The Pfaffl method of relative quantification was used to convert the resulting threshold cycle data for each sample to relative fold change information [5]. TFII-I mRNA levels were normalized to GAPDH mRNA levels.

SUPPLEMENTAL REFERENCES

1. **Cheriyath, V. and A.L. Roy.** 2000. Alternatively spliced isoforms of TFII-I. Complex formation, nuclear translocation, and differential gene regulation. J Biol Chem **275**:26300-26308.
2. **Tatham, M.H., M.S. Rodriguez, D.P. Xirodimas, and R.T. Hay.** 2009. Detection of protein SUMOylation in vivo. Nat Protoc **4**:1363-1371.
3. **Vink, E.I., M.A. Yondola, K. Wu, and P. Hearing.** 2012. Adenovirus E4-ORF3-dependent relocalization of TIF1alpha and TIF1gamma relies on access to the Coiled-Coil motif. Virol **422**:317-325.
4. **Evans, J.D. and P. Hearing.** 2003. Distinct roles of the Adenovirus E4 ORF3 protein in viral DNA replication and inhibition of genome concatenation. J Virol **77**:5295-5304.
5. **Pfaffl, M.W.** 2001. A new mathematical model for relative quantification in real-time RT-PCR. Nucleic Acids Res **29**:e45.
